# Cancer survivors' experiences with conversations about work‐related issues in the hospital setting

**DOI:** 10.1002/pon.5529

**Published:** 2020-10-19

**Authors:** Amber D. Zegers, Pieter Coenen, Mirjam van Belzen, Vivian Engelen, Carol Richel, Desiree J. S. Dona, Allard J. van der Beek, Saskia F. A. Duijts

**Affiliations:** ^1^ Department of Public and Occupational Health, Amsterdam UMC Vrije Universiteit Amsterdam, Amsterdam Public Health Research Institute Amsterdam The Netherlands; ^2^ Dutch Federation of Cancer Patients Organizations (Nederlandse Federatie van Kankerpatiëntenorganisaties, NFK) Utrecht The Netherlands; ^3^ Dutch Breast Cancer Organization (Borstkankervereniging Nederland, BVN) Utrecht The Netherlands; ^4^ Department of Human Resources Radboud university medical center Nijmegen The Netherlands; ^5^ Netherlands Comprehensive Cancer Organization (Integraal Kankercentrum Nederland, IKNL) Utrecht The Netherlands

**Keywords:** cancer, cancer care, communication, employability and cancer, information needs, oncology

## Abstract

**Objective:**

Early access to work‐related psychosocial cancer care can contribute to return to work of cancer survivors. We aimed to explore: (a) the extent to which hospital healthcare professionals conduct conversations about work‐related issues with cancer survivors, (b) whether cancer survivors experience these conversations as helpful, and (c) the possible financial implications for cancer survivors of (not) discussing their work early on.

**Methods:**

The Dutch Federation of Cancer Patient Organizations developed and conducted a cross‐sectional online survey, consisting of 27 items, among cancer survivors in the Netherlands.

**Results:**

In total, 3500 survivors participated in this study (71% female; mean age (SD) 56 (11) years). Thirty‐two percent reported to have had a conversation about work‐related issues with a healthcare professional in the hospital. Fifty‐four percent indicated that this conversation had been helpful to them. Conversations about work‐related issues took place more frequently with male cancer survivors, those aged 55 years or below, those diagnosed with gynecological, prostate, breast, and hematological or lymphatic cancer, those diagnosed ≤2 years ago, or those who received their last treatment ≤2 years ago. There was no statistically significant association between the occurrence of conversations about work‐related issues and experiencing the financial consequences of cancer and/or its treatment as burdensome.

**Conclusions:**

Although conversations about work‐related issues are generally experienced as helpful by cancer survivors, early access to work‐related psychosocial cancer care in the hospital setting is not yet systematically offered.

## 1 BACKGROUND

In the Netherlands, approximately 118 500 people are diagnosed with cancer annually, of whom 40% to 50% are of working age at diagnosis.^1^ Due to improved strategies to detect and treat various forms of cancer, the 5‐year survival rate is currently 65% across all ages and between 66% and 86% for those of working age.^2^ These statistics indicate that life with and beyond cancer is increasingly becoming a reality for a substantial proportion of the Dutch population. Work is often part of that reality and an emotional and/or financial necessity for many.^3^, ^4^ While about two‐thirds of working‐age cancer survivors (CSs)^5^ are able to return to work (RTW) within 2 years post diagnosis,^4^, ^6^ many experience long‐term physical, cognitive, and/or psychosocial limitations that hinder sustainable work participation.^7^, ^8^ We view cancer survivorship as “a process that begins at the moment of diagnosis and continues through the balance of life.”^5^ ^(p. 235)^

While the personal and societal advantages of being able to work after cancer are well‐known, access to work‐related psychosocial cancer care in the hospital setting is a relatively new topic in the international literature. In a Swedish sample, Söderman et al showed that initiating a conversation about work (ie, providing advice, support, and encouragement regarding RTW) early in the cancer trajectory can increase CSs' ability to RTW.^9^ Qualitative studies, such as those by Kennedy et al (UK),^10^ Stergiou‐Kita et al (Canada),^11^ and Maunsell et al (Canada)^12^ found that CSs have unmet needs regarding RTW guidance. Lastly, in a systematic review of studies conducted in European CSs, Paltrinieri et al found that work‐related support provided by healthcare professionals was positively associated with CSs' work participation.^13^ Conversely, either not addressing work at an early stage or encouraging patients to stay sick‐listed can contribute to longer sickness absence duration and increased financial difficulties in CSs.^13^

Currently, it is unknown to what extent CSs are met in their needs for work‐related guidance from physicians and other professionals in the hospital setting. Prior qualitative research has shown that few CSs reported having received useful advice from their cancer care team regarding RTW.^14^ Moreover, Bains et al reported that the limited offering of work‐related guidance by healthcare professionals was due to professionals' lack of knowledge of, and available resources on, work‐related consequences of cancer treatments.^15^

In the Netherlands, as in several other countries, general and occupational healthcare are organized in separate systems.^16^, ^17^ Whereas general healthcare professionals perform curative healthcare tasks, occupational healthcare professionals mainly provide sick leave assessments and reintegration guidance.^18^ The availability and accessibility of hospital‐based work‐related psychosocial cancer care varies per hospital and is often not covered by standard healthcare insurance. These shortcomings presumably contribute to a relatively low number of work‐related conversations between CSs and healthcare professionals in the Netherlands.

In this study, we aimed to explore: (a) the extent to which hospital healthcare professionals conduct conversations about work‐related issues with CSs, (b) whether CSs experience these conversations as helpful, and (c) the possible financial implications for CSs of (not) discussing their work early on.

## 2 METHODS

### 2.1 Study sample and procedures

This study was conducted by the Dutch Federation of Cancer Patient Organizations (NFK), an entity that unites 19 cancer patient organizations in the Netherlands. Data were collected through a national, cross‐sectional survey using “Survey Monkey.”^19^ The online survey was available for two weeks, from the end of February until the beginning of March 2019. A link to the survey was distributed via social media and e‐mailed to members of various cancer patient organizations and NFK's patient panel. The survey was aimed at CSs who were employed at time of diagnosis.

This study was reviewed by the Medical Ethics Review Committee of VU University Medical Center (2020.239) and found to be exempt from the Medical Research involving Human Subjects Act (WMO). In accordance with the General Data Protection Regulation (EU) 2016/679, respondents in this study were informed of the NFK's privacy policy. Recent articles presented a similar approach, using NFK data.^20^, ^21^

### 2.2 Survey development and content

The online survey was designed by NFK and consisted of 27 questions: 26 quantitative questions and 1 open answer question (Table S1). The survey started with a question to identify respondents who have (had) cancer. Respondents who did not select “I have (had) cancer” were excluded from further analyses. Thereafter, respondents were asked to report their gender, year of birth, and highest completed level of education. The remaining 23 questions were organized into six themes (Table S1). Data were collected and analyzed anonymously and stored securely.^22^

### 2.3 Statistical analyses

As this study was designed to be explorative, no minimum sample size was estimated a priori. Of 4556 participants who started the questionnaire, 3504 completed it. Four participants were excluded due to indecipherable answers or duplicate survey entries. Thus, 3500 participants were included for the current analyses.^23^ Descriptive statistics were obtained, i.e., percentages for nominal variables and mean and SD, as well as median and interquartile range (IQR) for continuous variables. Chi‐squared tests were used to answer the research questions. Answer categories “I don't know” and/or “Not applicable” were excluded from Chi‐squared analyses. Respondents who were retired at diagnosis, and were not otherwise employed, were also excluded from Chi‐squared analyses. For all analyses, *P‐*values ≤.05 were considered statistically significant. All analyses were performed using IBM SPSS Statistics version 25.^24^

## 3 RESULTS

Mean age of respondents was 56 years (SD = 11) and 71% was female (Table 1). Most respondents were diagnosed with breast cancer (38%) or hematological or lymphatic cancer (19%). The majority of respondents was treated in a teaching hospital (39%). Median time since diagnosis was 4 years (IQR = 6), and median time since last treatment was 2 years (IQR = 5). Nearly half of respondents had a fixed employment contract at time of survey completion (46%), and 8% was on (partial) sick leave from their work.

**TABLE 1:** Characteristics of study population (n *=* 3500)

	n (%)	
Gender, female	2482 (71)	
Age, mean (SD)	56 (11)	
Educational level^a^		
Low	272 (8)	
Medium	1457 (42)	
High	1727 (49)	
Other	44 (1)	
Type of cancer		
Breast	1327 (38)	
Hematological or lymphatic	666 (19)	
Colon	375 (11)	
Prostate	206 (6)	
Melanoma or skin	182 (5)	
Gynecological	143 (4)	
Lung	103 (3)	
Bladder	94 (3)	
Other	404 (11)	
Years since most recent cancer diagnosis (median (IQR))	(4 (6))	
Mean (SD)	6 (6)	
≤2 years ago	1906 (56)	
>2 years ago	1531 (44)	
Years since most recent cancer treatment (median (IQR))	(2 (5))	
Mean (SD)	4 (6)	
≤2 years ago	1906 (55)	
>2 years ago	1531 (45)	
Type of hospital		
Academic	1130 (32)	
Teaching	1369 (39)	
General	940 (27)	
Other	58 (2)	
Employment situation at time of diagnosis and at time of survey^b^		
Fixed employment contract	2418 (71)	1529 (46)
Temporary employment contract	283 (8)	151 (5)
Entrepreneur	393 (12)	338 (10)
Flex work	63 (2)	47 (1)
Unpaid work or volunteer work	129 (4)	173 (5)
Informal care	81 (2)	79 (2)
Job seeker	73 (2)	87 (3)
(Partly) on sick leave (work)	62 (2)	264 (8)
(Partly) on sick leave (Social Security Agency)	30 (1)	129 (4)
(Partly) work disabled	111 (3)	664 (20)
Retired	65 (2)	467 (14)
Not looking for a job due to education	39 (1)	13 (0)
Not looking for a job due to other reasons	39 (1)	49 (1)
Financial consequences of cancer (treatment)		
Yes	2082 (60)	
No	1269 (36)	
Don't know/N.A.	149 (4)	
Type of financial consequences^b^		
Lowered income	1810 (90)	
Increased healthcare costs	1367 (68)	
Financial consequences are experienced as a problem		
Never	556 (27)	
Sometimes	1036 (50)	
Often	314 (15)	
Always	177 (8)	

Abbreviations: IQR, interquartile range; SD, standard deviation.

^a^ Low = ISCED 0, 1, 2; medium = ISCED 3, 4; high = ISCED 5, 6, 7, 8.^23^

^b^ Multiple answers possible.

### 3.1 Conversations about work‐related issues

Nearly one‐third of respondents (n *=* 992, 32%) indicated that a healthcare professional within the hospital had discussed the work‐related consequences of cancer and/or its treatment with them. Respondents who indicated that work was discussed, most often reported that this was done by clinical nurse specialists, case managers, physician assistants (n *=* 523, 55%), physicians or medical specialists (n *=* 508, 53%), social workers or psychologists (n *=* 307, 32%), and/or occupational physicians specialized in oncology (n *=* 54, 6%) (multiple answers possible). Work‐related issues were discussed most often during treatment (n *=* 547, 57%) and/or follow‐up (n *=* 510, 53%) and least often around diagnosis (n *=* 327, 34%) (multiple answers possible).

Male CSs (35%) had a conversation about work‐related issues more often than female CSs (31%) (*P* ≤ .05). CSs aged 55 years or below (35%) had such a conversation more often than CSs aged above 55 (30%) (*P* ≤ .01). The occurrence of these conversations did not differ significantly by educational level (*P* = .25). While CSs diagnosed with gynecological (40%), prostate (36%), breast (34%), and hematological or lymphatic cancer (33%) reported to have had conversations about work‐related issues most often, these conversations took place least often in CSs diagnosed with lung cancer (23%). CSs who received their most recent diagnosis ≤2 years ago (37%) had a conversation about work‐related issues more frequently than CSs whose most recent diagnosis was >2 years ago (30%) (*P* ≤ .001). Similarly, CSs who received their last treatment ≤2 years ago (35%) reported these conversations to have taken place more often than CSs who received their last treatment >2 years ago (29%) (*P* ≤ .001). Occurrence of conversations about work‐related issues did not differ by hospital type (*P* = .95) (Table 2).

**TABLE 2:** Factors associated with the occurrence of a conversation about work‐related issues in the hospital

Did a healthcare professional in the hospital discuss work‐related consequences of cancer and/or its treatment with you?	n (%)		*χ* ^2^ (*df*)
	Yes	No	
Gender			
Male	309 (35)	571 (65)	*χ* ^*2*^ (1) = 4.6*
Female	683 (31)	1513 (69)	n = 3076
Age			
Age ≤ 55 years	518 (35)	974 (65)	*χ* ^*2*^ (1) = 8.1**
Age > 55 years	474 (30)	1110 (70)	n = 3076
Educational level			
Low	62 (28)	161 (72)	*χ* ^*2*^ (2) = 2.8
Medium	416 (33)	831 (67)	n = 3035
High	500 (32)	1065 (68)	*P* = .25
Type of cancer			
Breast	407 (34)	792 (66)	*χ* ^*2*^ (8) = 16.9*
Hematological or lymphatic	188 (33)	381 (67)	n = 3076
Colon	87 (27)	229 (73)	
Prostate	63 (36)	114 (64)	
Melanoma or skin	44 (26)	126 (74)	
Gynecological	52 (40)	77 (60)	
Lung	21 (23)	70 (77)	
Bladder	24 (31)	54 (69)	
Other	106 (30)	241 (70)	
Years since most recent cancer diagnosis			
≤2 years ago	331 (37)	562 (63)	*χ* ^*2*^ (1) = 13.4***
>2 years ago	661 (30)	1522 (70)	n = 3076
Years since most recent cancer treatment			
≤2 years ago	595 (35)	1114 (65)	*χ* ^*2*^ (1) = 11.8***
>2 years ago	382 (29)	938 (71)	n = 3029
Type of hospital			
Academic	313 (32)	677 (68)	*χ* ^*2*^ (3) = 0.4
Teaching	392 (33)	803 (67)	n = 3073
General	269 (32)	566 (68)	*P =* .95
Other	17 (32)	36 (68)	

Abbreviation: df, degrees of freedom.

^*^ *P =* ≤.05.

^**^ *P =* ≤.01.

^***^ *P =* ≤.001.

### 3.2 Cancer survivors' experiences and needs regarding conversations about work‐related issues

Of the CSs who had a conversation about work‐related issues, the majority indicated that this had been helpful to them (n *=* 537, 54%), 35% stated (n *=* 344) that it had been somewhat helpful, and 11% stated that it had not been helpful (n *=* 111). Further, 22% (n = 780) felt a need to discuss work with a healthcare professional. In general, female CSs (30%) expressed this need more often than males (20%) (*P* ≤ .001). Furthermore, CSs ≤55 years (35%) expressed this need more frequently than older CSs (20%) (*P* ≤ .001). CSs with high educational levels (30%) indicated this need more often than CSs with medium (24%) or low educational levels (22%) (*P* ≤ .01). CSs diagnosed with gynecological cancer (32%) reported the need for a conversation most frequently, followed by breast (30%), hematological or lymphatic (29%), and lung cancer (28%). CSs diagnosed with bladder (18%), colon (17%), and prostate cancer (11%) least often indicated a need for such a conversation (*P* ≤ .001). CSs who received their most recent diagnosis ≤2 years ago (34%) expressed the need for a conversation about work‐related issues more often than CSs with a less recent diagnosis (24%) (*P* ≤ .001). Lastly, CSs who received their most recent cancer treatment ≤2 years ago (31%) expressed this need more often than CSs who received their most recent treatment ≥2 years ago (21%) (*P* ≤ .001) (Table 3).

**TABLE 3:** Factors associated with needs for a conversation about work‐related issues in the hospital

Do you feel the need to discuss work‐related consequences of cancer and/or its treatment with a healthcare professional in the hospital?	n (%)		*χ* ^*2*^ (*df*)
	Yes	No	
Gender			
Male	177 (20)	690 (80)	*χ* ^*2*^ (1) = 26.2*
Female	603 (30)	1433 (70)	n = 2903
Age			
Age ≤ 55	477 (35)	883 (65)	*χ* ^*2*^ (1) = 87.7*
Age > 55	303 (20)	1240 (80)	n = 2903
Educational level			
Low	45 (22)	162 (78)	*χ* ^*2*^ (2) = 13.9*
Medium	285 (24)	900 (76)	n = 2870
High	440 (30)	1038 (70)	
Type of cancer			
Breast	330 (30)	767 (70)	*χ* ^*2*^ (8) = 53.9*
Hematological or lymphatic	162 (29)	391 (71)	n = 2903
Colon	54 (17)	258 (83)	
Prostate	18 (10)	153 (90)	
Melanoma or skin	38 (24)	121 (76)	
Gynecological	35 (32)	75 (68)	
Lung	23 (28)	59 (72)	
Bladder	15 (18)	68 (82)	
Other	105 (31)	231 (69)	
Years since most recent cancer diagnosis			
≤2 years ago	277 (34)	529 (66)	*χ* ^*2*^ (1) = 31.9*
>2 years ago	503 (24)	1594 (76)	n = 2903
Years since most recent cancer treatment			
≤2 years ago	500 (31)	1101 (69)	*χ* ^*2*^ (1) = 35.2*
>2 years ago	267 (21)	986 (79)	n = 2854

Abbreviation: *df*, degrees of freedom.

^*^ *P =* ≤.001.

### 3.3 Financial consequences of (not) discussing work early on in the hospital setting

Of all CSs, 60% (n *=* 2082) reported that cancer and/or its treatment has had financial consequences, 90% (n *=* 1810) of which indicated lowered income, and 68% (n = 1367) of which indicated increased healthcare costs (multiple answers were possible). Further, 27% stated that the reported financial consequences have never been a problem to them, whereas for 50%, 15%, and 8%, this was sometimes, often, or always a problem, respectively (Table 1). No statistically significant association between the occurrence of work‐related conversations and experiencing financial consequences as burdensome was found (*P* = .15) (Table 4). We did not further explore these associations for specific subgroups (eg, age groups or diagnosis categories).

**TABLE 4:** Associations between the occurrence of conversations about work‐related issues and financial consequences

Did a healthcare professional in the hospital discuss work‐related consequences of cancer and/or its treatment with you?	n (%)		*χ* ^*2*^ (*df*)
	Yes	No	
Have the financial consequences of cancer and/or its treatment been a problem for you?			
Never	181 (36)	322 (64)	*χ* ^*2*^ (3) = 5.3
Sometimes	307 (32)	646 (68)	n = 1904
Often	82 (29)	203 (71)	*P =* .15
Always	48 (29)	115 (71)	

Abbreviation: *df*, degrees of freedom.

## 4 DISCUSSION

Healthcare professionals in the Dutch hospital setting did not systematically discuss work‐related consequences of cancer and/or its treatment (ie, 68% of CSs reported that they had not been engaged in such a conversation). Work‐related psychosocial cancer care in the hospital setting is more often provided to specific survivors (ie, male CSs, those aged ≤55, those diagnosed with gynecological, prostate, breast, hematological, or lymphatic cancer, those diagnosed ≤2 years ago, and those who received treatment ≤2 years ago). The majority of CSs who had had a conversation about work‐related issues indicated that it had been helpful. Finally, there was no statistically significant association between the occurrence of conversations about work‐related issues and experiencing the financial consequences of cancer and/or its treatment as burdensome.

Based on these findings, we conclude that there is room for improvement in hospital‐based work‐related psychosocial cancer care in the Netherlands. Our results showed that only 32% of CSs had been engaged in a conversation about work‐related issues. The frequency of such conversations did not differ by hospital type. As described previously, a relatively low occurrence of conversations about work‐related issues can be expected within the Dutch hospital setting, due to the separation of general and occupational healthcare in the Netherlands.^16^, ^18^ To compare, in a study by Söderman et al,^25^ 80% of Swedish breast CSs reported to have had a conversation about work‐related issues with a healthcare professional in the hospital within one year post surgery. Taking only breast CSs into account, our results still showed a marked difference compared to these Swedish findings (ie, 34% vs 80%, respectively). CSs in our sample had, on average, completed their treatment a longer time ago than CSs in Söderman et al,^25^ which might have contributed to these differences. Furthermore, Dutch and Swedish social security systems are differently organized, for example, in Sweden, reintegration guidance is part of hospital‐based care paid by healthcare insurance rather than part of occupational healthcare paid by companies in the Netherlands.^26^

Our results showed that male CSs had a conversation about work‐related issues more frequently than female CSs. Within the Dutch family composition, the family's income is often largely dependent on the male's salary. Men commonly work full‐time and women often part‐time (ie, 27% of women vs 72% of men worked full‐time in 2019).^27^ Part‐time work, flexible working hours, and the decision to stop working after cancer diagnosis therefore might be culturally viewed as more acceptable in women than in men. However, female CSs expressed a need to discuss the work‐related consequences of cancer and/or its treatment more often than male CSs. Although a large percentage of women in the Netherlands work part‐time, a national increase in working women has been observed over the years, which might contribute to higher needs for work‐related support in female compared to male CSs.^28^ Additionally, female CSs might report the need for such a conversation more often than male CSs simply because they receive such conversations less often.

Our findings showed that conversations about work‐related issues take place more often with CSs aged ≤55 years, than with CSs aged >55. One possible explanation for this is that healthcare professionals might view paid employment as more relevant for younger CSs than for older CSs. Older age has been identified as a predictive factor in early retirement for CSs^29^ but does not preclude older CSs from wanting to work or having a need for work‐related guidance. To illustrate, in our sample, 20% of CSs >55 years indicated a need for a conversation about work‐related issues. Considering that retirement ages are rising and that employment can contribute to CSs' health‐related quality of life,^30^ it is pertinent that healthcare professionals pay attention to the work‐related needs of CSs of all ages.

Additionally, we found that CSs whose last cancer diagnosis was ≤2 years ago reported conversations about work‐related issues more frequently than CSs whose last cancer diagnosis was >2 years ago. However, 24% of CSs whose most recent cancer diagnosis was >2 years ago indicated a need for such a conversation. Potentially, these CSs have not received timely work‐related guidance in the hospital. Alternatively, extensive treatment and/or follow‐up trajectories or other health‐related priorities might have excluded them from earlier guidance. We found that CSs with gynecological, prostate, breast, hematological, or lymphatic cancer most frequently reported to have been engaged in a conversation about work‐related issues in the hospital. These diagnoses have relatively high 5‐year survival rates,^1^ making paid work a relevant topic for these CSs.

Characteristics of CSs who indicated the need to discuss work‐related issues most frequently were similar to characteristics of CSs who reported that a conversation about work‐related issues had actually taken place (ie, being aged ≤55 years, having been diagnosed ≤2 years ago, being diagnosed with gynecological, breast, hematological, or lymphatic cancer, having received one's most recent treatment ≤2 years ago). It might be that CSs with these characteristics are better able to articulate their needs and actively seek guidance from hospital‐based healthcare professionals than CSs who do not express such needs.

Finally, our findings showed that there was no statistically significant association between the occurrence of work‐related conversations and experiencing the financial consequences of cancer and/or its treatment as burdensome. This finding is not in line with previous research, showing that failing to address work early on can contribute to financial difficulties for CSs.^9^, ^25^ It could be that our sample was too heterogeneous to detect meaningful differences. Additional research is needed to assess whether early discussion of employment issues in the hospital setting could mitigate the pervasiveness of financial consequences for CSs.

## 5 CONCLUSIONS

Whereas conversations about work‐related issues were generally experienced as helpful by CSs, only 32% of Dutch CSs reported to have had such a conversation in the hospital setting. These conversations were initiated more often with specific CSs (ie, males, those aged ≤55, those with specific cancer diagnoses, those diagnosed ≤2 years ago, and those who received treatment ≤2 years ago). Our findings suggest that early access to work‐related psychosocial cancer care in the hospital setting is not yet systematically offered. Professionals in this sector have a unique opportunity to contribute to CSs' rehabilitation and societal participation.

### 5.1 Study limitations

This study was based on a large‐scale nation‐wide survey in the Netherlands, which aimed to describe CSs' experiences regarding conversations about work‐related issues in Dutch hospital settings. Being the first of its kind in the Netherlands, and one of the first studies on this topic internationally, our findings contribute to scientific research in the field of access to work‐related psychosocial cancer care.

Several limitations should be noted. First, the composition of our sample complicates generalization of our results to more diverse CSs populations. When comparing our results to national data of CSs (aged 18‐67) in 2019,^1^ we found that our sample had a slightly higher age (mean 56 (SD 11) vs 53 (11) years), had received their diagnosis and/or treatment more recently (diagnosis 6 (6) vs 10 (8) years ago; treatment 4 (6) vs 6 (5) years ago), and were more often female (71% vs 61%). However, main cancer diagnosis (breast cancer) and treatment hospital (teaching hospital) were comparable. Second, highly educated CSs were overrepresented in our sample, compared to the general CSs population.^21^ To conclude, while there are some discrepancies between our sample and national averages in 2019, similarities can be found as well. Therefore, extrapolation of our findings to the Dutch CSs population should be done with caution.

Third, this study was conducted in the context of the Dutch social security and healthcare system, which hampers generalization to countries with different systems. Fourth, as median time post diagnosis was 4 years, recall bias might have been present. Fifth, as this study was cross‐sectional, we cannot infer causality from our findings. Finally, the questionnaire that the NFK used was not validated, which may complicate international comparison and weaken the quality of the presented evidence.

### 5.2 Clinical implications

Hospital‐based work‐related guidance can contribute to CSs' ability to RTW.^9^, ^25^ Moreover, labor participation can contribute to CSs' quality of life as well as their mental and physical well‐being.^30^ Thus, hospital‐based healthcare professionals have a unique opportunity to contribute to CSs' rehabilitation and societal participation, by making (return to) work a regular topic of discussion early on. Yet, our results showed that work‐related consequences of cancer and/or its treatment are not systematically discussed in the Dutch hospital setting.

We recommend that healthcare professionals, in their conversations about work‐related issues, take into account factors such as age, gender, cancer diagnosis, and time since most recent diagnosis and treatment, alongside other predictive factors of sustainable work participation in CSs. Multidisciplinary treatment teams should reach a consensus on who to put forward as first point of contact regarding work‐related issues. Hospital‐based healthcare professionals, for example, occupational therapists,^31^ can meaningfully prepare CSs for RTW by enhancing CSs' self‐efficacy regarding cognitive and physical side‐effects of cancer treatment. Reintegration planning and supporting communication with the workplace (eg, occupational physician) should be outsourced by healthcare professionals to community‐based professionals, for example, reintegration consultants, in a collaborative effort to bridge the gap between medical (after)care and societal reintegration.

## CONFLICT OF INTEREST

The authors have no conflict of interest to disclose.

## Supporting information


**TABLE S1** NFK survey themes.

## Data Availability

The data that support the findings of this study are available from NFK. Restrictions apply to the availability of these data, which were used under license for this study. Data are available from and with the permission of NFK.
