# Supplementary material for: Cancer survivors' experiences with conversations about work‐related issues in the hospital setting
Source: Psychooncology. 2020 Oct 19;30(1):27–34. doi: 10.1002/pon.5529 (PMC7894286; doi:10.1002/pon.5529)
Supplement: Supplementary file 1 — TABLE S1 NFK survey themes. [file PON-30-27-s001.docx]

**SUPPORTING INFORMATION I**

**Supplementary information about the NFK questionnaire**

Three out of 27 questions were scored on a 10-point scale ranging from 1 (very dissatisfied) to 10 (very satisfied). All other questions were followed by multiple-choice answer options, date selection options or open-ended answer fields.

**Themes within the NFK questionnaire**

The questionnaire contained six themes, four of which were included in the current analyses: diagnosis and treatment, employment situation, attention for consequences at work, and financial consequences (Table S1).

**TABLE S1:** NFK survey themes

| Theme | Items | Content | Included in this study yes/no |
| --- | --- | --- | --- |
| 1. Diagnosis and treatment | 4 | Date and type of most recent cancer diagnosis, date of most recent cancer treatment, type of treatment hospital. | Yes |
| 2. Employment situation | 2 | Employment status at time of diagnosis and at time of survey participation. | Yes |
| 3. Consequences at work | 5 | Changes in work situation due to cancer and/or its treatment, the agent of this change (e.g., the cancer survivor, occupational physician, or supervisor), and satisfaction with the adjusted work situation. | No |
| 4. Attention for consequences at work | 4 | Needs for discussing work-related consequences of cancer and/or its treatment with hospital-based health care professionals, past occurrence(s) of such conversations, which hospital-based health care professionals were involved in such conversations, timing and helpfulness of these conversations. | Yes |
| 5. Occupational physician | 5 | Received guidance from occupational/insurance physicians, constituents and completeness of this guidance, and satisfaction with guidance. | No |
| 6. Financial consequences | 3 | Extent to which respondents experienced financial consequences of the cancer diagnosis and/or treatment trajectory, type and pervasiveness of these consequences. | Yes |
